# Memory network plasticity after temporal lobe resection: a longitudinal functional imaging study

**DOI:** 10.1093/brain/awv365

**Published:** 2016-01-09

**Authors:** Meneka K. Sidhu, Jason Stretton, Gavin P. Winston, Andrew W. McEvoy, Mark Symms, Pamela J. Thompson, Matthias J. Koepp, John S. Duncan

**Affiliations:** ^1^ 1 Department of Clinical and Experimental Epilepsy, UCL Institute of Neurology, Queen Square, London WC1N 3BG, UK; ^2^ 2 Epilepsy Society MRI Unit, Chesham Lane, Chalfont St. Peter SL9 0RJ, Buckinghamshire, UK; ^3^ 3 MRC Cognition and Brain Science Unit, Chaucer Road, Cambridge, CB2 7EF, UK

**Keywords:** temporal lobe epilepsy, memory encoding, functional MRI, anterior temporal lobe resection

## Abstract

Anterior temporal lobe resection can control seizures in up to 80% of patients with temporal lobe epilepsy. Memory decrements are the main neurocognitive complication. Preoperative functional reorganization has been described in memory networks, but less is known of postoperative reorganization. We investigated reorganization of memory-encoding networks preoperatively and 3 and 12 months after surgery. We studied 36 patients with unilateral medial temporal lobe epilepsy (19 right) before and 3 and 12 months after anterior temporal lobe resection. Fifteen healthy control subjects were studied at three equivalent time points. All subjects had neuropsychological testing at each of the three time points. A functional magnetic resonance imaging memory-encoding paradigm of words and faces was performed with subsequent out-of-scanner recognition assessments. Changes in activations across the time points in each patient group were compared to changes in the control group in a single flexible factorial analysis. Postoperative change in memory across the time points was correlated with postoperative activations to investigate the efficiency of reorganized networks. Left temporal lobe epilepsy patients showed increased right anterior hippocampal and frontal activation at both 3 and 12 months after surgery relative to preoperatively, for word and face encoding, with a concomitant reduction in left frontal activation 12 months postoperatively. Right anterior hippocampal activation 12 months postoperatively correlated significantly with improved verbal learning in patients with left temporal lobe epilepsy from preoperatively to 12 months postoperatively. Preoperatively, there was significant left posterior hippocampal activation that was sustained 3 months postoperatively at word encoding, and increased at face encoding. For both word and face encoding this was significantly reduced from 3 to 12 months postoperatively. Patients with right temporal lobe epilepsy showed increased left anterior hippocampal activation on word encoding from 3 to 12 months postoperatively compared to preoperatively. On face encoding, left anterior hippocampal activations were present preoperatively and 12 months postoperatively. Left anterior hippocampal and orbitofrontal cortex activations correlated with improvements in both design and verbal learning 12 months postoperatively. On face encoding, there were significantly increased left posterior hippocampal activations that reduced significantly from 3 to 12 months postoperatively. Postoperative changes occur in the memory-encoding network in both left and right temporal lobe epilepsy patients across both verbal and visual domains. Three months after surgery, compensatory posterior hippocampal reorganization that occurs is transient and inefficient. Engagement of the contralateral hippocampus 12 months after surgery represented efficient reorganization in both patient groups, suggesting that the contralateral hippocampus contributes to memory outcome 12 months after surgery.

## Teaser

Memory decrements are the main neurocognitive complication of anterior temporal lobe resection for refractory temporal lobe epilepsy. Sidhu *et al.* report dynamic changes in memory-encoding networks three and twelve months post-surgery compared to preoperative measures and to healthy controls, and describe the neural correlates of successful post-operative memory reorganisation.

## Graphical abstract

**Figure:**
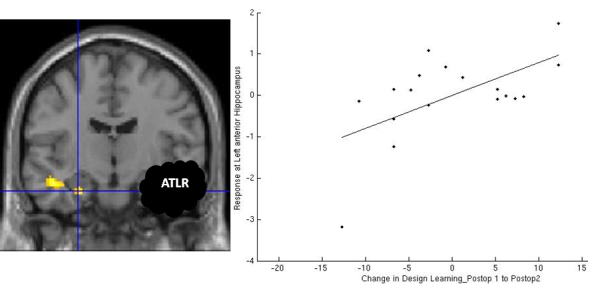
Memory decrements are the main neurocognitive complication of anterior temporal lobe resection for refractory temporal lobe epilepsy. Sidhu *et al.* report dynamic changes in memory-encoding networks three and twelve months post-surgery compared to preoperative measures and to healthy controls, and describe the neural correlates of successful post-operative memory reorganisation.

## Introduction

Temporal lobe epilepsy (TLE) is associated with widespread cognitive deficits with material-specific episodic memory impairment being most commonly described; particularly verbal memory loss in patients with left TLE and visual in those with right TLE. More recently, a move away from the material-specific model describes task-related disruption with both verbal and visual deficits seen across patients with left and right TLE (Gleissner *et al.*, 2002; Flugel *et al.*, 2006; Glikmann-Johnston *et al.*, 2008; Saling, 2009). Functional MRI studies have shown temporal and extra-temporal reorganization within memory encoding networks across both verbal and visual domains in individuals with both left and right TLE (Dupont *et al.*, 2002; Bonelli *et al.*, 2010; Alessio *et al.*, 2013; Sidhu *et al.*, 2013).

Up to 80% of patients with TLE achieve remissions of at least 12 months after anterior temporal lobe resection (ATLR) (de Tisi *et al.*, 2011). Although deterioration of both verbal and visual episodic memory has been described as a consequence of surgery, verbal memory decline after dominant ATLR remains the most consistent finding, occurring in up to 30% of patients.

Functional MRI has been used to predict patients at risk of memory decline after ATLR. The greater the activation within the ‘to-be-resected’ anterior medial temporal lobe, the greater the verbal and visual decline after left and right ATLR, respectively (Richardson *et al.*, 2004; Powell *et al.*, 2008; Bonelli *et al.*, 2010; Binder, 2011) in keeping with the hippocampal adequacy model of memory outcome after ATLR (Chelune, 1995). Recently, our group showed that activation of the posterior hippocampus preoperatively was related to memory preservation postoperatively (Bonelli *et al.*, 2013). Correspondingly, the extent of hippocampal resection is an important determinant of postoperative memory function and has important implications on surgical planning (Baxendale *et al.*, 2000; Alpherts *et al.*, 2008; Schramm, 2008).

Plasticity in the memory-encoding networks within the medial temporal lobe after ATLR has only been described in a few studies (Cheung *et al.*, 2009; Korsnes *et al.*, 2009; Bonelli *et al.*, 2013) with one reporting extra-temporal postoperative memory network plasticity within a small frontal region (Maccotta *et al.*, 2007). Previously, using a material-specific word encoding task our group showed that memory reorganization to the posterior hippocampus preoperatively was predictive of preserved verbal memory function after left ATLR. Greater postoperative reorganization to the ipsilateral posterior hippocampus correlated with worse verbal memory 4 months after resection indicating that effective reorganization to the posterior left hippocampus did not occur in the initial postoperative months (Bonelli *et al.*, 2013). This study was confined to the temporal lobes.

In controls, although memory encoding test-retest studies have shown stable hippocampal activations at least 3 months after initial scanning (Atri *et al.*, 2011; Putcha *et al.*, 2011), task similarities may incur practice effects and altered memory-encoding strategies in healthy controls (Siders *et al.*, 2006) leading to differential engagement of the frontal lobes (Fletcher and Henson, 2001). To date, no studies have described quantitative postoperative network plasticity changes compared to changes in controls imaged across similar time intervals. We previously reported plasticity in the working memory network 3 and 12 months after anterior temporal lobe resection relative to changes in controls imaged across similar time intervals (Stretton *et al.*, 2014).

This study represents a separate cohort of patients to those reported by Bonelli *et al.* (2013). We extended the field of view to achieve whole cortex coverage. Previously, in this cohort of patients, we described the preoperative functional anatomy of verbal and visual memory encoding in patients with right TLE and those with left TLE (Sidhu *et al.*, 2013, 2015*a*). Subsequently, we described a method of using verbal memory functional MRI to predict verbal memory decline following ATLR (Sidhu *et al.*, 2015*b*). In this study, we first investigated dynamic medial temporal and extra-temporal reorganization of verbal and visual memory-encoding networks 3 and 12 months after ATLR in patients with left and right TLE compared to healthy controls; and second, used a material-specific event-related analysis to study successful memory encoding with correlations with postoperative changes in neuropsychological performance to determine the efficiency of postoperative changes (Sidhu *et al.*, 2013).

## Materials and methods

### Subjects

We studied 36 patients with medically refractory TLE [17 left: median age 32 years, interquartile range (IQR) 26–45; 19 right: median age 40, IQR 22–48]. All underwent presurgical evaluation and surgery at the National Hospital for Neurology and Neurosurgery, London. All patients had structural MRI at 3 T including quantification of hippocampal volumes and T_2_ relaxation times preoperatively and at 3 and 12 months after surgery. In the left TLE group, 11 patients had unilateral hippocampal sclerosis, two anterior temporal cavernomas, two dysembryoplastic neuroepithelial tumours and in two patients no lesion was identified. In the right TLE group, 10 had unilateral hippocampal sclerosis, four had dysembryoplastic neuroepithelial tumours, one had non-specific high signal of the parahippocampal gyrus and in four patients no structural lesion was seen.

Prolonged interictal and ictal EEG-video telemetry confirmed ipsilateral seizure onset zones in all patients. All patients received antiepileptic medication and spoke fluent English. All patients underwent standard *en bloc* temporal lobe resections (which involved opening of the temporal horn, followed by resection of the hippocampus with a posterior resection margin at the mid-brainstem level), functional MRI and detailed neuropsychometry preoperatively and at 3 and 12 months after surgery. International League Against Epilepsy (ILAE) postoperative seizure outcome (Wieser *et al.*, 2001) and changes in antiepileptic medication 3 and 12 months after surgery were recorded (Table 1).

**Table 1:** Clinical details of patients with left and right TLE

	Left TLE (*n = *17)	Right TLE (*n = *19)
Handedness (left/right)	2/15	3/16
Language dominance (right/left/bilateral)	1/16/0	1/17/1
Median age at onset of epilepsy (IQR), years	14 (7–24)	14 (9–18)
Median duration of epilepsy (IQR), years	14 (7–25)	18 (8.5–32)
Median duration to first postoperative scan, Postoperative 1 (IQR), months	3.5 (3.2–3.7)	3.7 (3.1–4.6)
Median duration to second postoperative scan, Postoperative 2 (IQR), months	12.6 (12.1–13.6)	12.9 (12.1–13.3)
AED change at 3 months	Nil	Nil
AED change at 12 months	6/17 one AED reduced or stopped	7/19 one AED reduced
	2/17 off AEDs	2/19 off AEDs
ILAE seizure outcome at 3 months	14/17 outcome 1–2	16/19 outcome 1–2
	3/17 outcome 3–5	2/19 outcome 3–4
		1/19 outcome 5
ILAE seizure outcome at 12 months	15/17 outcome 1–2	16/19 outcome 1–2
	2/17 outcome 4	2/19 outcome 4
		1/19 outcome 5

AED = anti-epileptic drug; ILAE = International League Against Epilepsy.

Fifteen healthy native English speaking matched controls [median age 40 (IQR: 30.5–49)] underwent memory functional MRI and neuropsychometry at three similar time intervals to patients. There was no significant age difference between controls and patients with left TLE and those with right TLE (*P* > 0.1). All participants were recruited between February 2010 and February 2012. All patients underwent ATLR before March 2012 and the final longitudinal neuropsychometry and functional MRI analyses were acquired by March 2013.

Handedness and language dominance were determined using a standardized questionnaire (Oldfield, 1971) and language functional MRI tasks (Powell *et al.*, 2006), respectively. Expressive language lateralization (LI) within an inferior and middle frontal gyrus mask created using the WFU PickAtlas in SPM8 (Maldjian *et al.*, 2003), was calculated (Bonelli *et al.*, 2010). A LI of ≥0.5 or <−0.5 was deemed strongly left or right lateralized, respectively (Table 1). This study was approved by the National Hospital for Neurology and Neurosurgery and the UCL Institute of Neurology Joint Research Ethics Committee. Written informed consent was obtained from all participants.

### Neuropsychological testing

All patients and controls underwent standardized cognitive assessments including measures of intellectual functioning; IQ (Nelson and O’Connell, 1978) and memory using the BIRT Memory and Information Processing Battery (BMIPB) verbal learning and design learning subtest (Coughlan, 2007), previously demonstrated as sensitive to the integrity of temporal structures (Baxendale *et al.*, 2006) at three time points; preoperatively, 3 months and 12 months after surgery in patients and three equivalent time intervals in controls. In the verbal learning task, participants are read a list of 15 words over five trials with recall tested after each trial. The sum of recalled words is the performance measure. A similar neuropsychological test in which an abstract design was presented was used to calculate design learning scores.

In patients and controls, preoperative scores were subtracted from second and third assessments as a measure of change in memory performance over time. Postoperative change at 3 and 12 months was correlated with functional activations pattern 3 and 12 months postoperatively. Clinically significant decline in verbal and visual memory was calculated using reliable change indices (Baxendale and Thompson, 2005); defined as decline of 12 points on the verbal learning task and 13 on the design learning task (90% confidence interval).

Change in verbal and design learning scores between preoperative and 3 and 12 months postoperative time points were correlated with changes in ipsilesional hippocampal volumes at the corresponding time points in patients. All statistical analyses were performed using PASW Statistics 18.0 (IBM).

### Magnetic resonance data acquisition

Studies were performed using a 3 T General Electric Excite HDx MRI scanner with a maximum gradient strength of 40 mTm^−1^ and slew rate 150 Tm^−1^s^−1^. For the functional MRI, gradient-echo echo planar images were acquired, providing blood oxygen level-dependent contrast. Each volume comprised 36 contiguous oblique axial slices, slice thickness 2.5 mm (0.3 mm gap), field of view 24 cm, matrix 96 × 96 interpolated to 128 × 128 during image reconstruction, in-plane resolution 2.5 mm × 2.5 mm, SENSE factor 2.5, echo time 25 ms, repetition time 2.75 s. The field of view covered the temporal and frontal lobes with slices aligned with the long axis of the hippocampus.

### Memory encoding paradigm

Visual (faces) and verbal stimuli (words) were visually presented during a single scanning session (Sidhu *et al.*, 2013). For faces, a combination of neutral and fearful non-famous faces unfamiliar to the subjects was used. For words, single concrete nouns and emotionally averse words (cancer, terrorist, famine) were used. All stimuli were presented on a magnetic resonance compatible screen viewed via a mirror. Each item was presented for 3 s in 60-s blocks. We used a different interstimulus interval (3 s) to our repetition time of 2.75 s to introduce jitter and ensure random sampling. Each block consisted of 10 faces (five fearful) and 10 words (two emotionally averse) followed by 15 s cross hair fixation. We presented a total of 10 blocks (100 faces and 100 words). Participants were explicitly instructed to memorize items for subsequent out-of-scanner recall. A deep encoding task (Craik, 2002), which involved a subjective decision on whether each stimulus was pleasant or unpleasant, using a joystick was performed.

Forty minutes after scanning, face and word recognition was tested separately in an out-of-scanner recognition task. In each recognition task, subjects were shown the same 100 items intermixed with an additional 50 novel faces/words as foils in random order at the same speed as items were displayed within the scanner.

A button box was used to indicate if items were remembered, familiar or novel. These responses were used to sort each item shown in the scanner to items remembered, familiar and forgotten. Recognition accuracy (%) was calculated for both faces and words (% true positive – % false positive).

An identical task with different words and faces was employed for the second and third scanning time points in both patients and controls.

### Data analysis

#### Preprocessing of preoperative functional MRI data

Analysis was performed using SPM8 (http://www.fil.ion.ucl.ac.uk/spm/). The imaging time series was realigned and normalized into standard anatomical space using a scanner-specific, high resolution whole brain EPI template created from 30 healthy controls, 15 patients with left hippocampal sclerosis and 15 patients with right hippocampal sclerosis. As part of the SPM programme for normalization, a two stage linear and non-linear registration method was used. Realigned and normalized images were smoothed with a Gaussian kernel of 8 mm full-width at half-maximum.

#### Preprocessing of postoperative functional MRI data

The baseline imaging time series of each patient was realigned using the mean image as a reference. Rigid body co-registration was used to co-register postoperative scans to the preoperative mean image; scans were then spatially normalized into standard space applying each subject’s preoperative spatial normalization parameters to the subject’s postoperative realigned and co-registered scans. All scans were then smoothed with a Gaussian kernel of 8 mm full-width at half-maximum.

### Event-related analysis

Event-related analyses on a blocked design experiment have been performed in memory studies (Richardson *et al.*, 2003; Powell *et al.*, 2007; Bonelli *et al.*, 2010). We compared the encoding-related responses for stimuli that were subsequently remembered versus stimuli that were subsequently forgotten or rated familiar. A two-level event-related random-effects analysis was used.

#### First level

For each subject, trial-specific delta functions were convolved with the canonical haemodynamic response function and its temporal derivative. Six regressors of interest for each of the event types, words remembered (WRem), words familiar (WFam), words forgotten (WFo), faces remembered (FRem), faces familiar (FFam) and faces forgotten (FFo) were created. Each subject’s movement parameters were included as confounds. Contrast images were created for each subject for word encoding [defined by (WRem) − (WFam + WFo)] and face encoding [defined by (FRem) − (FFam + FFo)] (Sidhu *et al.*2013). This was performed for each of the three scanning sessions in patients and controls; denoted Preoperative, Postoperative 1 and Postoperative 2 for word and face encoding. These images were used for the second level analysis.

#### Second level

##### One sample *t*-test

At the second level, one sample *t*-tests were performed in all controls, and patients with left and right TLE to model the group effect for the contrasts (WRem) − (WFam + WFo) and (FRem) − (FFam + FFo) at the three scanning time points. For controls, a within subject ANOVA was performed to study longitudinal changes across the three scanning sessions. Changes in patients relative to changes in controls were investigated in a flexible factorial analysis (Stretton *et al.*, 2014).

##### Flexible factorial analysis

To investigate the relationship between pre- and postoperative change in memory in the individual patient groups compared to changes in test-retest in controls at the same time intervals, we used a mixed ANOVA using a flexible factorial design (Glascher and Gitelman, 2008). The difference between controls and left TLE, and controls and right TLE groups were analysed in different flexible factorial sessions as this model only allows two groups to be compared at a time. Faces and words analyses were performed separately. For each flexible factorial, a factor of group with two levels (controls and left TLE or right TLE) and a factor of condition with three levels (Preoperative, Postoperative 1 and Postoperative 2) was specified. The relevant contrast images for each subject for each of the three conditions were entered allowing the investigation of a Group × Condition interaction for the contrasts of interest. Differences in activations across scanning sessions were compared between TLE patients and controls (for example word encoding left TLE > Controls: Postoperative 1 > Preoperative, Postoperative 2 > Preoperative, Postoperative 2 > Postoperative 1, Preoperative > Postoperative 1, Preopertive > Postoperative 2, Postoperative 1 > Postoperative 2).

Thus, we modelled the changes in activations at the three time points and between the groups, while controlling for between subjects and between group variance in a single model.

### Correlations with memory performance

A simple regression model of individual change in verbal learning and design learning as continuous regressors in an analysis of covariance (ANCOVA) against verbal and visual blood oxygen level-dependent subsequent memory group activations was used to assess brain activations corresponding to change in memory scores between the time points in patients with left TLE and right TLE.

### Statistical thresholds for reporting all functional MRI results

All differences in activations within and between groups and correlations are shown at a threshold of *P* < 0.001, uncorrected.

Previous studies have shown changes in medial temporal lobe memory functional MRI activations as a consequence of surgery (Cheung *et al.*, 2009; Bonelli *et al.*, 2013) with one suggesting that the anterior and posterior medial temporal lobes are differently engaged (Bonelli *et al.*, 2013). As such this region was of *a priori* interest and medial temporal lobe activations are shown corrected for multiple comparisons, family-wise error (FWE) using a small volume correction within a sphere diameter of 10 mm (FWE *P* < 0.05) unless otherwise stated (Bonelli *et al.*, 2013; Sidhu *et al.*, 2013).

### Parameter estimates

As one of the main aims of this study was to study the dynamic changes of medial temporal activations, we calculated the parameter estimates for word and face encoding preoperatively and at 3 and 12 months after surgery within the ipsilateral posterior and contralateral hippocampus where we previously showed preoperative activations (Sidhu *et al.*, 2013). Parameter estimates were extracted from the activation maps.

## Results

### Postoperative neuropsychology

#### Left TLE

Controls performed significantly better than patients with left TLE at all three time points. As a group, there was a non-significant decline in verbal learning from Preoperative to Postoperative 1 and Preoperative to Postoperative 2 (paired sample *t*-test, *P* > 0.05) and a significant decline in design learning from Preoperative to Postoperative 1 (paired sample *t*-test, *P* < 0.05) (Table 2).

**Table 2:** Neuropsychometry and behavioural measures across the three time points in control subjects and patients with left and right TLE

	Recognition accuracy words			Recognition accuracy faces			Verbal learning			Design learning		
	T1	T2	T3	T1	T2	T3	T1	T2	T3	T1	T2	T3
Controls	77.9	82.3	78.6	28.6	30.8	36.1	58.6	57.3	61.9	39.4	37.9	38.5
	(7.8)	(9.6)	(11.9)	(13)	(11.6)	(13)	(7.1)	(5.5)	(6.9)	(5.9)	(6.5)	(5.6)
Left TLE	60.2^a^	48.7^a^	45.8^a^	20.2	22.5	22.3^a^	47.5^a^	43.2^a^	44.8^a^	37.2	32.8	34.1^a^
	(15.9)	(20)	(19.4)	(10.8)	(13.5)	(10.1)	(10.7)	(14.5)	(12.4)	(4.9)	(7.5)	(7.1)
Right TLE	63.2	55.4^a^	49.5^a^	15.3^a^	9.5 ^a^^,^^b^	11.6^a^^,^^b^	47.9^a^	43.1^a^	44.6^a^	32.1^a^	28.5^a^	31.2^a^
	(18.2)	(21.8)	(25)	(8.5)	(9.5)	(12.3)	(11.2)	(11.7)	(14.1)	(8.7)	(10)	(9.4)

^a^Controls performed significantly better than patient group indicated *P* < 0.01.

^b^Right TLE performed significantly worse than left TLE *P* < 0.01.

In patients, T1 = preoperative; T2 = 3 months postoperatively; T3 = 12 months postoperatively. All values are reported as mean (standard deviation).

Three months postoperatively, 29.4% (5/17) and 17.6% (3/17) of patients with left TLE declined significantly in verbal learning and design learning, respectively.

Twelve months postoperatively, compared to Preoperative, 29.4% (5/17) and 5.9 % (1/17) of patients with left TLE declined significantly in verbal learning and design learning, respectively.

#### Right TLE

Controls performed better than patients with right TLE across both verbal and design learning tasks across all time points. Patients with right TLE showed a non-significant decline in design learning scores from Preoperative to Postoperative 1 and Preoperative to Postoperative 2 (paired sample *t*-test, *P > *0.05) and a significant decline in verbal learning between Preoperative and Postoperative 1 (paired sample *t*-test, *P* < 0.05) (Table 2).

Three months postoperatively, 15.8% (3/19) and 21% (4/19) of patients with right TLE declined significantly in design learning and verbal learning, respectively.

Twelve months postoperatively, 10.5% (2/19) and 21%(4/19) of patients with right TLE declined significantly compared to Preoperative in design learning and verbal learning, respectively. All patients with left TLE and those with right TLE who declined significantly in verbal learning at 3 and 12 months postoperatively were left hemisphere dominant for language (LI > 0.5). From Postoperative 1 to Postoperative 2 there was a non-significant improvement in both verbal learning and design learning in both patient groups (paired sample *t*-test, *P > *0.05), Table 2.

### Hippocampal volumes and correlation with change in memory

There was no significant difference between the ipsilesional and contralesional preoperative hippocampal volumes in patients with left TLE and those with right TLE (independent sample *t*-test *P > *0.05). Three months postoperatively, patients with left TLE had significantly larger posterior hippocampal residual volumes compared to patients with right TLE (independent sample *t*-test *P* < 0.05). Patients with left TLE showed significant decline in left hippocampal remnant volume from 3 to 12 months postoperatively, paired sample *t*-test, *P* = 0.019 [left hippocampal volume in patients with left TLE, in mm^3^: Preoperative 1.98 (0.6), Postoperative 1 0.79 (0.7), Postoperative 2 0.70 (0.6); right hippocampal volume in patients with right TLE 2.27 (0.4), Postoperative 1 0.23 (0.1), Postoperative 2 0.20 (0.1)].

There was no correlation of change in the ipsilateral remnant hippocampal volume from 3 to 12 months postoperatively with change in verbal learning and design learning at the corresponding time points in either left TLE or right TLE groups, Pearson correlation coefficient <0.3, two-tailed *t*-test *P* > 0.05.

### Functional MRI results

#### Behavioural: recognition accuracy

At baseline, controls had significantly better word recognition accuracy than patients with left TLE and face recognition accuracy than patients with right TLE (independent sample *t*-test *P* < 0.05).

Patients with left TLE showed a significant decline in recognition accuracy for words from Preoperative to Postoperative 1 and Preoperative to Postoperative 2 (paired sample *t*-test *P > *0.05) (Table 2). At Postoperative 1 and Postoperative 2, patients with right TLE also showed significantly worse word recognition accuracy compared to controls.

In patients with right TLE there was a significant decline in recognition accuracy for faces from Preoperative to Postoperative 1 and Preoperative to Postoperative 2 (paired sample *t*-test *P* < 0.05). No significant change in either word or face recognition accuracy was seen from Postoperative 1 to Postoperative 2 in patients with left TLE or those with right TLE.

A repeated measures ANOVA was performed for verbal learning, design learning, word and face recognition accuracy. Only for face recognition accuracy was there a significant effect of side of surgery on outcome, with significant decline in patients with right TLE but not left TLE (*P* = 0.001).

#### Baseline

For details see Supplementary Table 1. On word encoding, controls activated the left anterior hippocampus, patients with left TLE the left posterior hippocampus. Patients with right TLE showed bilateral anterior hippocampal activations. Controls and patients with right TLE showed right extra-temporal activations whereas patients with left TLE showed bilateral extra-temporal activations.

On face encoding, controls showed right anterior hippocampal activations; patients with left TLE showed bilateral posterior hippocampal activations; and patients with right TLE showed right posterior and left anterior hippocampal activations. Patients with left TLE showed left extra-temporal, whereas patients with right TLE showed bilateral extra-temporal activations. Controls showed right extra-temporal activations only at a lower threshold (*P* = 0.01, uncorrected).

Exploring baseline differences between controls and patients, patients with left TLE showed reduced predominantly left hemispheric and increased right hemispheric activations across word and face subsequent memory compared to controls. Changes in patients with right TLE were more bilateral compared to controls (Supplementary Table 1). Differences in group activations were similar to our previous study where preoperative reorganization was described (Sidhu *et al.*, 2013).

### Controls: longitudinal changes

#### Word encoding

Controls showed increased activations in the left inferior frontal gyrus at time points two (T2) and three (T3) compared to the first scanning session (T1). No changes in medial temporal activations, or areas of reduced activation were seen (Supplementary Table 2).

#### Face encoding

Controls showed increased left inferior frontal and orbitofrontal cortex activations at T2 and T3, respectively compared to T1. Reduced activations were noticed within the right hippocampus and orbitofrontal cortex at T2 with reduced activations seen within the right hippocampus at T3 compared to T1 (Supplementary Table 2).

### Left TLE changes compared to changes in controls

See Table 3 for details.

**Table 3:** Longitudinal changes in word and face encoding activations in patients with left TLE and right TLE relative to changes in encoding activations in controls

Region	Coordinate	Z-score	*P*-value	Region	Coordinate		Z-score		*P*-value
**Left TLE > Controls word encoding**				**Left TLE > Controls face encoding**					
**Postoperative 1 > Preoperative**									
R orbitofrontal C	36 34−14	3.79	0.000	R anterior HC	26−16 −14		3.84		0.001*
R anterior HC/PHG	24 −16 −18	3.43	0.003*	R mid temp G	48 −4 −28		4.11		0.000
R anterior PHG	16 −4 −22	2.71	0.026*	L posterior HC	−28 −26 −10		3.4		0.004*
				R postcentral G	60 −4 18		3.34		0.000
**Postoperative 2 > Preoperative**									
R inf frontal G	46 24 6	2.68	0.004	N/S					
R anterior HC	30 −14 −18	1.81	0.035						
**Postoperative 2 > Postoperative 1**									
R mid frontal G	34 10 34	2.87	0.002	R inf frontal G	−40 8 16		3.12		0.001
**Postoperative 1 < Preoperative**									
N/S				L inf frontal G	−42 10 18		3.09		0.001
**Postoperative 2 < Preoperative**									
L mid frontal G	−48 24 28	2.64	0.004	L inf frontal G	−50 22 16		3.09		0.001
L anterior HC	−28 −18 −16	1.88	0.03	L mid temp G	−56 −66 18		3.07		0.001
L posterior HC	−28 −30 −4	1.71	0.044						
**Postoperative 2 < Postoperative 1**									
L posterior PHG	−14 −36 −10	2.06	0.02	R posterior HC	22 −38 4		3.75		0.001*
L posterior HC	−30 −32 −6	1.82	0.03	L posterior HC	−18 −32 −2		3.44		0.004*
				R anterior PHG	26 2 −32		3.92		0.001*
**Right TLE > Controls word encoding**				**Right TLE > Controls face encoding**					

**Postoperative 1 > Preoperative**									
N/S				L posterior HC		−22 −34 −2		2.07	0.069*
				L anterior fusiform gyrus/PHG		−32 −14 −24		2.10	0.018
**Postoperative 2 > Preoperative**									
N/S				N/S					
**Postoperative 2 > Postoperative 1**									
L anterior HC	−16 −10 −22	2.35	0.044*	L postcentral G		−38 −34 46		2.97	0.001
R MFG	24 −6 52	2.97	0.001						
**Postoperative 1 < Preoperative**									
R anterior hippocampus	39 −22 −14(36 −12 −20)	2.61	0.025*	L inf parietal L		−38 −38 50		3.54	0.000
R hippocampal body	28 −24 −8	2.26	0.050*	R postcentral G		36 −26 46		3.32	0.001
**Postoperative 2 < Preoperative**									
L med OFC	0 42 −8	3.39	0.000	L anterior HC		−26 −20 −16		2.28	0.048*
L anterior cingulum	−5 18 26	3.21	0.001	R posterior HC		28 −34 0		1.72	0.043
L sup temporal pole	−34 10 −24	3.06	0.001						
**Postoperative 2 < Postoperative 1**									
L posterior HC	−24 −36 6	2.73	0.026*	L posterior HC		−20 −34 0		2.77	0.019*
L med OFC	2 32 −14	3.11	0.001	R posterior HC		30 −28 −2		2.11	0.060*

*Medial temporal activations are shown corrected for multiple corrections, FWE, *P* < 0.05 within a 10-mm diameter sphere. Postoperative 1 = 3 months postoperatively; Postoperative 2 = 12 months postoperatively; L = left; R = right; Sup = superior; Med = medial; Mid = middle; Inf = inferior; G = gyrus; OFC = orbitofrontal cortex; HC = hippocampus; PHG = parahippocampal gyrus; Temp = temporal; C = cortex; N/S = no significant activations.

#### Word encoding

Three months postoperatively, patients with left TLE showed a significant increase in right hemispheric activations within the right anterior hippocampus, parahippocampal gyrus and orbitofrontal cortex relative to changes in controls.

Twelve months after surgery, compared to baseline, there were reduced left-sided activations within the middle frontal gyrus and posterior hippocampus with an increase in right-sided activations within the inferior frontal gyrus and right anterior hippocampus relative to changes in controls.

From 3 to 12 months after surgery, there were significantly increased right middle frontal gyrus activations and reduced left posterior hippocampal and parahippocampal gyrus activations relative to changes in controls.

### Parameter estimates of word encoding activations in the medial temporal lobe in patients with left TLE

**Figure 1:**
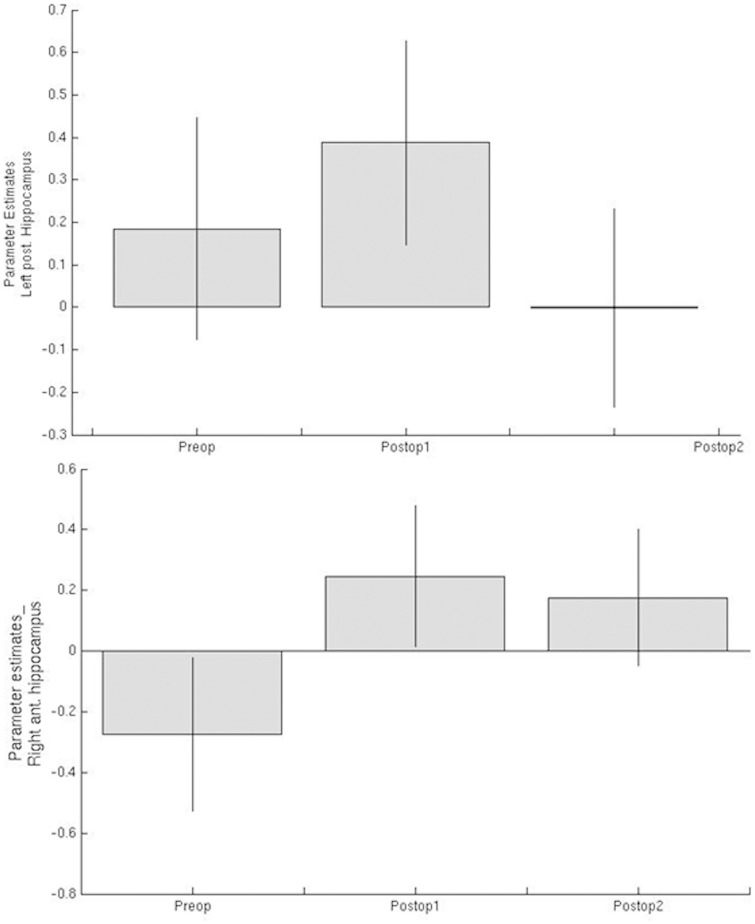
**Parameter estimates of word encoding medial temporal lobe activations preoperatively (Preop) and at 3 (Postop1) and 12 months (Postop2) after anterior temporal lobe resection in patients with left TLE**.

Preoperative left posterior hippocampus word-encoding activations in patients with left TLE were sustained 3 months postoperatively, but this significantly declined 12 months after surgery. Increased activation was seen within the right hippocampus 3 months postoperatively and this was sustained 12 months postoperatively (Fig. 1).

#### Face encoding

Three months postoperatively, patients with left TLE showed significantly reduced left inferior frontal gyrus and middle frontal gyrus activations with increased right-sided activations within the anterior hippocampus, middle temporal gyrus, postcentral gyrus and the left posterior hippocampus relative to changes in controls.

Twelve months postoperatively, compared with preoperatively, there were significantly reduced left inferior and middle frontal gyrus activations with increased right middle temporal gyrus activations relative to controls.

From 3 to 12 months after surgery, there was a significant increase in right inferior frontal gyrus activations and reduced activations within the posterior hippocampi bilaterally relative to changes in controls.

### Efficiency of 12-month postoperative change in left TLE

**Figure 2:**
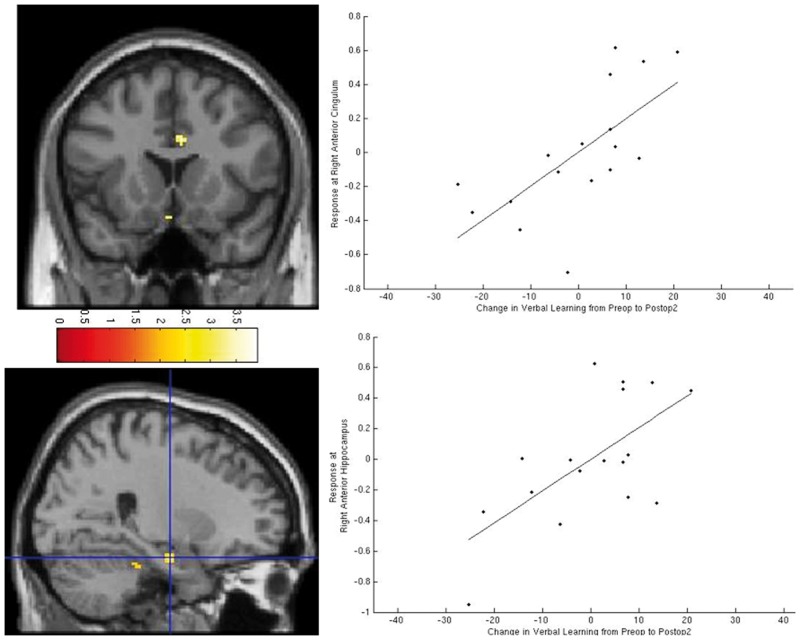
**Correlation of improvement in verbal learning 12 months postoperatively (Postop2) compared with preoperatively (Preop) in patients with left TLE.** The images show significant correlation of right anterior cingulum and anterior hippocampus activations with improvements in verbal learning 12 months postoperatively.

Improvement in verbal learning 12 months postoperatively compared to preoperatively correlated significantly with right anterior hippocampal and right extra-temporal activation within the anterior cingulum and parietal lobe (Supplementary Table 3 and Fig. 2). Improvement in design learning from 3 months postoperatively compared with preoperatively, correlated with right anterior cingulum activations at 3 months.

### Right TLE changes compared to changes in control subjects

See Table 3 for details.

#### Face encoding

Three months postoperatively, patients with right TLE showed reduced left inferior parietal lobule and right post-central gyrus activations and increased left posterior hippocampal activations relative to changes in controls.

Twelve months postoperatively there was a reduction in left anterior hippocampal and right posterior hippocampal activation compared to preoperatively.

From 3 to 12 months postoperatively, there was a significant decrease in left and right posterior hippocampal and left anterior hippocampal activation.

### Parameter estimates of face encoding activations in the middle temporal lobe in patients with right TLE

**Figure 3:**
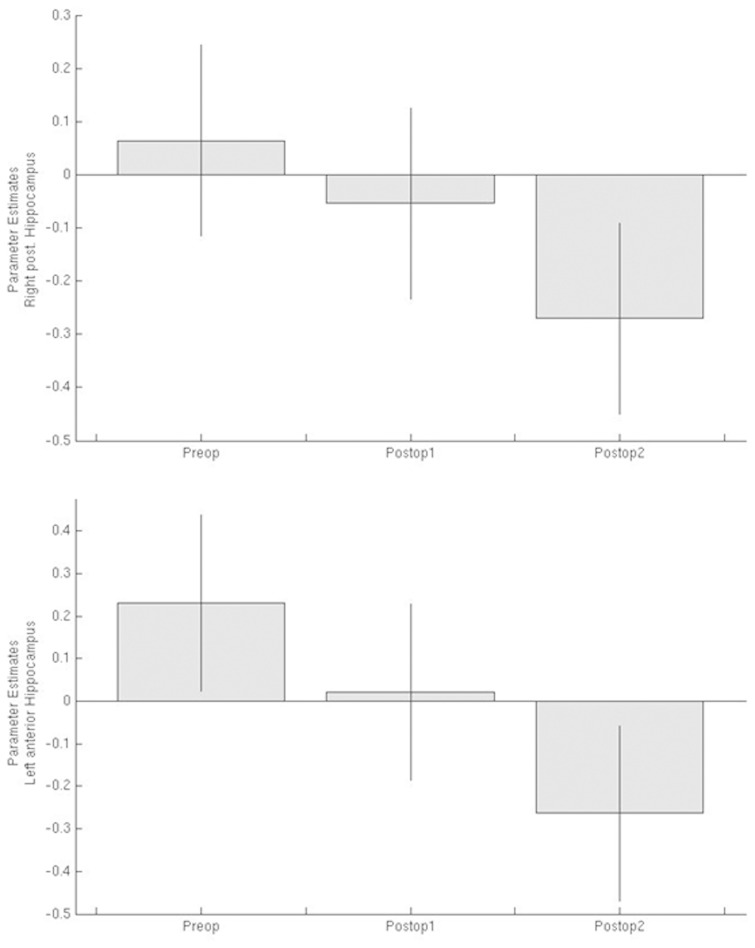
**Parameter estimates of face encoding medial temporal lobe activations preoperatively and at 3 (Postop 1) and 12 months (Postop 2) after anterior temporal lobe resection in patients with right TLE**.

Patients with right TLE showed activation within the left anterior hippocampus and right posterior hippocampus preoperatively. At Postoperative 1, there was a reduction in both activations with a further reduction in activation 12 months after surgery (Fig. 3).

#### Word encoding

Three months postoperatively, patients with right TLE had reduced right anterior hippocampal activations at word encoding relative to changes in controls.

Twelve months postoperatively, there were reduced left hemispheric activations within the left medial orbitofrontal cortex, anterior cingulum and superior temporal pole relative to changes in controls.

From 3 to 12 months postoperatively, there were increased left anterior hippocampal and right middle frontal gyrus activations with reduced activations within the left posterior hippocampus.

### Efficiency of 12-month postoperative change in right TLE

**Figure 4:**
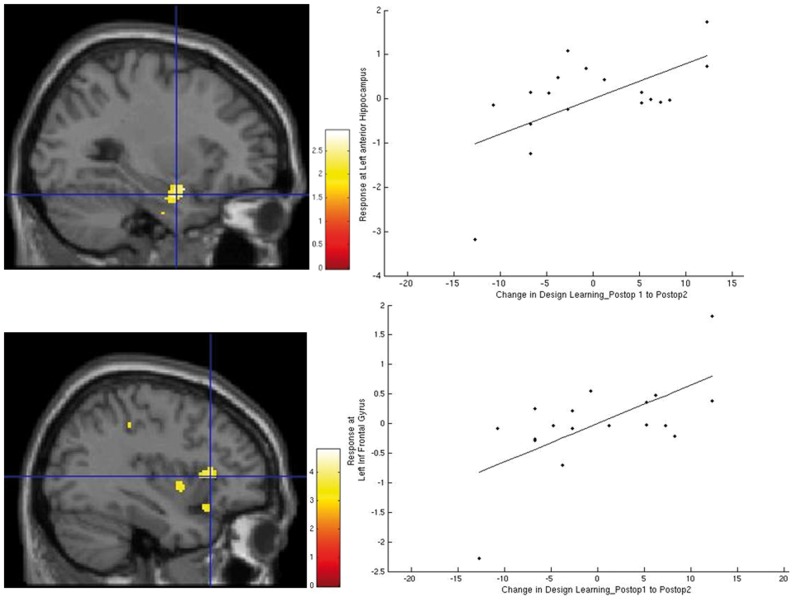
**Correlation of improvement in design learning 12 months postoperatively in patients with right TLE.** The images show significant correlation of left inferior frontal gyrus, insula, orbitofrontal cortex and anterior medial temporal activations 12 months postoperatively with improvements in design learning 12 months postoperatively. Postop1 = 3 months postoperatively; Postop2 = 12 months postoperatively.

Improvement in design learning 12 months postoperatively compared to 3 months postoperatively correlated significantly with left anterior medial temporal (amygdala and hippocampus) and left extra-temporal activations within the orbitofrontal cortex, inferior frontal gyrus and insula 12 months postoperatively (Supplementary Table 3 and Fig. 4).

Improvement in verbal learning 12 months postoperatively compared to preoperatively, correlated significantly with left anterior hippocampal and left orbitofrontal cortex activation 12 months postoperatively (Supplementary Table 3).

The increased left posterior hippocampal face encoding activation 3 months postoperatively compared to preoperatively correlated significantly with decline in design learning in patients with right TLE (Supplementary Fig. 1). Decline in verbal 3 months postoperatively compared to preoperatively correlated with left posterior hippocampal activation 3 months postoperatively (Supplementary Table 3).

## Discussion

This study examined the effects of temporal lobe resection on verbal and visual memory encoding networks in patients with left TLE and those with right TLE, relative to longitudinal changes in controls, 3 and 12 months after ATLR. Next, a voxel-by-voxel whole brain analysis of activations 3 and 12 months postoperatively were correlated with change in verbal and visual memory to further investigate brain areas involved in improvement or decline in memory functions after ATLR.

Controls showed increased left frontal activations at the second and third scanning sessions compared to the first, on both word and face encoding. No medial temporal lobe changes were seen on word encoding, while on face encoding, reduced right hippocampal activations were seen at both the second and third scanning sessions.

Preoperatively, both patients with left and right TLE showed ipsilesional posterior hippocampal activation during word and face encoding, respectively. Postoperative changes are summarized in Table 4. Patients with left TLE showed increased contralateral right anterior hippocampal and right extra-temporal activation both at 3 and 12 months after surgery on word and face encoding, with a concomitant reduction in left frontal activation 12 months postoperatively. Right anterior hippocampal activation 12 months postoperatively correlated significantly with improved verbal learning in patients with left TLE from preoperative to 12 months postoperatively, representing efficient plasticity. Extra-temporal activations within the right anterior cingulum correlated with improved verbal learning 12 months postoperatively. Three months postoperatively, the preoperative left posterior hippocampal activation was sustained during word encoding, increased during face encoding but was significantly reduced 12 months postoperatively for both word and face encoding.

**Table 4:** Summary of longitudinal changes in word and face encoding activations in patients with left TLE and right TLE relative to changes in control subjects

	Increases at 3 months	Decreases at 3 months	Increases at 12 months	Decreases at 12 months
Activation changes in LTLE patients relative to controls				
Verbal memory	Right OFC, ant HC and PHG	N/S	Right ant HC (versus preop) and right MFG (versus 3 months)	L MFG (versus preop)
				L post HC and PHG (versus 3 months)
Visual memory	Right ant HC, MTG, PCG	Left IFG	Right IFG (versus 3 months)	L IFG (versus preop)
	Left post HC			BL posterior HC (versus 3 months)
**Activation changes in RTLE patients relative to controls**				
Verbal memory	N/S	R HC (area of resection)	Right MFG, Left ant HC (versus 3months)	Left OFC, ACC (versus preop)
				Left OFC and post HC (versus 3 months)
Visual memory	Left ant PHG and post HC	Left IPL	Left PCG (versus 3 months)	BL post HC (versus 3 months)
		Right PCG		

Ant = anterior; BL = bilateral; OFC = orbitofrontal cortex; HC = hippocampus; PHG = parahippocampal gyrus; N/S = no significant activations; MTG = middle temporal gyrus; MFG =middle frontal gyrus; ACC anterior cingulum; IPL = inferior parietal lobule; PCG = post-central gyrus.

Patients with right TLE showed an increase in left anterior hippocampal activation on word encoding from 3 to 12 months postoperatively. On face encoding, left anterior hippocampal activations were present preoperatively and 12 months postoperatively. These left anterior hippocampal and orbitofrontal cortex activations correlated with improvements in both design and verbal learning, implying efficient plasticity in these structures 12 months after surgery.

Three months postoperatively, patients with right TLE showed increased left posterior hippocampal activations on face encoding. This was not involved in successful memory formation as it correlated with a decline in design learning 3 months postoperatively.

### Memory outcome after temporal lobe resection

Behavioural measures showed significant decline in verbal and visual recognition accuracy in patients with left and right TLE 12 months after surgery, respectively. Although neuropsychometry showed a non-significant decline in verbal learning and a significant decline in design learning in patients with left TLE as a group, a greater proportion of patients with left TLE had significant verbal memory decline (29%) compared to visual memory decline (6%) 12 months postoperatively.

Patients with right TLE showed a non-significant decline in design learning as a group and significant decline in verbal learning. A greater proportion of right TLE (11%) patients showed decline in non-verbal memory compared to patients with left TLE (6%). Interestingly, 21% of patients with right TLE also showed significant verbal memory impairment 12 months postoperatively. Historically, material-specific deficits with verbal deficits after left temporal resections and visual deficits after right temporal resection (Milner, 1966) have been described. One explanation may be that left- and right-sided resection patients were compared to each other and not with memory in healthy controls. More recently, verbal memory decline after non-dominant resections has been increasingly described. In a recent study of 124 patients with hippocampal sclerosis, laterality was not identified as a risk factor (Murphy and Cook, 2010). This is in keeping with the task-specific concept proposed by Saling in which patients with left and right TLE were equally impaired on certain verbal learning measures but not others (Saling *et al.*, 1993; Saling, 2009).

In both patient groups, there was no significant change in either verbal or visual memory from 3 to 12 months after surgery. This is in keeping with longitudinal studies on patients up to 5 (Baxendale *et al.*, 2012) and 10 years (Engman *et al.*, 2006) after surgery. On an individual level however, several factors such as preoperative memory, postoperative seizure outcome, mood and medication change play a significant role in memory outcome after surgery.

### Longitudinal functional MRI changes in control subjects

Controls showed stable medial temporal lobe activations on word encoding but reduced right hippocampal activations on face encoding on retesting. This may be explained by the ‘novelty encoding’ hypothesis, which suggests that the encoding of online information into long-term memory is influenced by its novelty and that novelty increases recognition performance (Tulving *et al.*, 1996). We presented black and white faces and although the faces were varied at the second and third scanning sessions, the ‘novelty’ effect may be lost. With word encoding there was less of a novelty effect to begin with as the words were known to subjects. We compared longitudinal changes in encoding activations in patients after anterior temporal lobe resection to longitudinal changes seen in controls to ensure changes reported were not simply due to repeat scanning.

### Plasticity in patients with left TLE

Irrespective of material type, patients with left TLE showed dynamic changes in both medial temporal lobe and frontal activations with increased contralateral right anterior hippocampal and right frontal activation both at 3 and 12 months after surgery on word and face encoding.

Three months postoperatively there was an increase in left posterior hippocampal activation for face encoding and less significantly on word encoding, which correlated with reduced design learning, implying inefficient recruitment of the posterior hippocampus 3 months after surgery. This is in keeping with our previous study where we showed increased left posterior hippocampal activation in a group of patients with left TLE on word encoding 4 months after surgery that correlated with a decline in postoperative memory (Bonelli *et al.*, 2013). In the current study, we extend this to show that posterior hippocampal activation 3 months postoperatively was transitory with significantly reduced posterior hippocampal activation from 3 to 12 months postoperatively for both word and face encoding.

One study to date examined extra-temporal memory encoding network plasticity after surgery in TLE patients but in a limited inferior frontal gyrus region of interest (Maccotta *et al.*, 2007). Pre- and postoperative signal change within this region was compared quantitatively in this word and face classification study. Reduced right frontal activation was found postoperatively during word encoding in patients with left TLE suggesting a more left lateralized network postoperatively. No differences were described on visual encoding. No medial temporal lobe activation changes were reported. Several patient and scanning factors may explain the difference from our results. Firstly, an implicit word and face classification task in which memorization was not encouraged was used. Greater activation has been shown with explicit tasks where memorization is encouraged, as in our paradigm. Second, a blocked design analysis in which subsequent memory was not investigated was used therefore it is not known if activations represent successful memory formation. Third, not all patients underwent ATLR as in our study. Some underwent selective amygdalohippocampectomy or lateral lesionectomy. No change in either word or face recognition was seen postoperatively in this small number of patients (eight left TLE) while we showed that patients with left TLE declined in the word recognition task. Finally, the postoperative time period varied with patients being scanned between 5 and 9 months after surgery. We showed that frontal changes in patients with left TLE were dynamic therefore a varied period of analysis may not capture the true nature of frontal lobe engagement at different stages.

### Plasticity in patients with right TLE

Patients with right TLE showed dynamic medial temporal lobe changes but showed less extra-temporal plasticity effects than patients with left TLE. This may be because patients with left TLE showed greater decline in both verbal and visual memory than patients with right TLE 3 months postoperatively. No increase in extra-temporal activations were seen 3 months postoperatively but, as in left TLE, increases in extra-temporal activations were seen 3 to 12 months postoperatively compared to controls; left frontal and left parietal on word and face encoding, respectively. Patients with right TLE also showed increased left posterior hippocampal activation 3 months postoperatively that correlated with decline in visual and verbal memory 3 months postoperatively, implying inefficient early postoperative recruitment. Increments in posterior hippocampal activation were transient, with relative suppression of this activation from 3 to 12 months postoperatively. Similar to left TLE, contralesional anterior medial temporal lobe activation reflected efficient plasticity 12 months postoperatively.

Bonelli *et al.* (2013) reported no significant change in medial temporal lobe activation on face encoding in patients with right TLE 4 months postoperatively. This could be because no patients with right TLE declined significantly in design leaning. Postoperative change in word encoding was not investigated. In the study by Macotta *et al.* (2007) detailed above, no postoperative change within an inferior frontal gyrus region of interest in patients with right TLE at both word and face encoding was seen. In all these aforementioned longitudinal studies, changes in reported activations were not reported relative to changes in controls.

### Efficiency of reorganized networks

In patients with right TLE, contralateral hemispheric activations within the left anterior middle temporal lobe and orbitofrontal cortex represented ‘efficient’ activations irrespective of material type 12 months after ATLR. The role of the orbitofrontal cortex in successful memory formation has been described in TLE patients (Sidhu *et al.*, 2013), healthy control subjects (Frey and Petrides, 2003) and in lesional studies (Meunier *et al.*, 1997). This may be due to its connections to limbic structures including the amygdala, hippocampus, temporal pole, entorhinal, perirhinal and parahippocampal cortices (Insausti *et al.*, 1987; Carmichael and Price, 1995; Lavenex *et al.*, 2002). Surgery, however, led to efficient engagement of the hemisphere contralateral to the resection. Similarly, in patients with left TLE, contralateral activations within the right medial temporal lobe and anterior cingulum represented efficient reorganization 12 months postoperatively. Preoperative efficient anterior cingulum activations in verbal memory formation in temporal lobe epilepsy have been previously described (Eliassen *et al.*, 2008; Sidhu *et al.*, 2013). We showed that this activation is also efficient 12 months postoperatively.

We previously reported dynamic changes in the working memory network in this cohort of patients (Stretton *et al.*, 2014). In healthy controls, the medial temporal lobes were deactivated during working memory tasks whereas left and right TLE patients failed to show ipsilesioanal medial temporal lobe deactivation. Three to 12 months after surgery, patients with left TLE showed greater contralesional right hippocampal deactivation that correlated with improved working memory performance. In concert with our findings, the contralesional hippocampus was efficiently engaged 12 months after surgery.

In the only other functional MRI study of changes 12 months postoperatively, Cheung *et al.* (2009) reported a similar pattern in which the contralateral medial temporal lobe played an efficient compensatory role in maintaining verbal and visual episodic memory 12 months after surgery. In this study a non-material specific scene encoding task was used and a block design was employed in a small number of TLE patients (nine left, eight right). Although more sensitive, blocked design analysis is less specific to activations that represent successful memory formation.

### Neurobiological implications

We showed efficient contralateral fronto-temporal recruitment 12 months postoperatively in both patient groups. These changes could represent a ‘release’ phenomenon with reversal of a functional or metabolic disruption. Functional imaging studies such as fluorodeoxyglucose-PET showed a normalization of glucose metabolism in the ipsilateral temporal cortex (Gogoleva *et al.*, 1997), inferior frontal lobe, thalamus and parietal lobe (Spanaki *et al.*, 2000; Takaya *et al.*, 2009) following temporal lobe surgery.

Structural imaging studies such as diffusion tensor imaging have also shown ipsilateral and contralateral structural recovery after surgery (Concha *et al.*, 2005; Thivard *et al.*, 2007; Yogarajah *et al.*, 2008; Winston *et al.*, 2014). Pfeuty *et al.* (2011) showed that structural recovery in the contralateral anterior hippocampus correlated with improvements in verbal and visual memory postoperatively, in keeping with our findings of efficient contralateral anterior hippocampal reorganization, irrespective of material type.

### Strengths and limitations

This is the first longitudinal memory functional MRI study to investigate postoperative plasticity at two time points after surgery relative to changes in controls imaged across similar time points. All patients underwent a homogenous surgical procedure as other surgical approaches may cause different cognitive outcomes (Helmstaedter, 2013). We studied patients with left TLE and patients with right TLE separately and reported dynamic changes across both verbal and visual memory encoding. We used a flexible factorial design of analysis that is a quantitative model that allowed the comparison of longitudinal changes in patients against longitudinal changes in controls, which enables controlling for between subject and between group variance in a single model.

Our study also has limitations. It is known that factors such as medication (Yasuda *et al.*, 2013; Wandschneider *et al.*, 2014) and mood (Flugel *et al.*, 2006) can impact significantly on cognition. Although no medication changes had been made 3 months postoperatively, 8/17 patients with left TLE and 9/19 patients with right TLE had at least one anti-epileptic drug reduced 12 months postoperatively. Drug and mood changes were not accounted for in our analysis. Importantly, there was no significant difference between the patient groups in the number of patients with drug reductions. Further interval studies between 3 and 12 months postoperatively are required to ascertain the temporal cadence of change in brain activations more accurately.

Although medial temporal activations were shown corrected for multiple corrections within an *a priori* region (small volume correction), changes in extra-temporal activations across the time points were reported at a more liberal threshold of 0.001 uncorrected. This threshold was chosen as firstly, we performed a whole brain event-related analysis which is more specific but less sensitive than blocked-design analyses. Next, changes in activation that we report represent not just longitudinal changes in patients across three time points but changes in patients relative to changes in controls across these time points, analysed in a single specific flexible factorial analysis for each patient group.

Anterior medial temporal lobe activations are highly susceptible to signal drop out and distortion. We used a parallel imaging factor (SENSE) of 2.5 and reduced our echo time to 25 ms, which helped with data quality as evidenced by significant medial temporal lobe activations. Future studies, however, may benefit from using dual echo methods of image acquisition that have recently been shown to be a superior imaging technique (Halai *et al.*, 2014).

In conclusion, dynamic postoperative changes occur in the memory encoding network in both left and right TLE patients across both verbal and visual domains. Three months after surgery, compensatory posterior hippocampal reorganization that occurs is transient. Engagement of the contralateral hippocampus 12 months after surgery represents efficient reorganization in both patient groups, suggesting that the contralateral hippocampus influences memory outcome 12 months after surgery. This is compatible with descriptions of functional recovery and structural plasticity in the contralateral medial temporal lobe after surgery.

## Supplementary Material

Supplementary Data

Supplementary Table 1

## Abbreviations

ATLR: anterior temporal lobe resection
TLE: temporal lobe epilepsy
