# Supplementary material for: Memory network plasticity after temporal lobe resection: a longitudinal functional imaging study
Source: Brain. 2016 Jan 9;139(2):415–30. doi: 10.1093/brain/awv365 (PMC4805088; doi:10.1093/brain/awv365)
Supplement: Supplementary Data [file awv365_supplementary_data.zip › brain-2015-00632-File008.pdf]

Region	Coordinate	Z score	P value	Region	Coordinate	Z score	P value
Word encoding				Face encoding			
Controls							
L Med OFC	-16 62 -4	3.02	0.001	R Mid Temp G	64 -6 -22	3.29	0.000
L Mid Frontal G	-40 12 34	2.99	0.001	R Inf OFC	42 34 -16	2.25	0.01
L Rolandic O	-42 -6 14	3.43	0.000				
Controls_ Medial temporal lobe							
L Ant HC	-24 0 -28	2.71	0.02*	R Ant HC	28 -14 -12	3.79	0.000*
				L Fusiform G	-24 -40 -12	3.37	0.000*
LTLE							
R Mid Frontal G/ Precentral G	54 -10 44	3.82	0.000	L Sup Temp G/ L Post Insula	-44-16 4	3.55	0.000
R Sup Temp G	70-34 2	3.19	0.001	L Med Frontal G	-4 14 -16	2.99	0.001
L Sup Frontal G	-18 2 46	3.18	0.001	L Post cingulum	-6 -42 14	3.1	0.001
L Orbitofrontal G	-8 66 -2	3.18	0.001				
L Fusiform	-22 -42 -16	3.17	0.001				
LTLE_Medial temporal lobe							
L Post HC	-32 -32 -6	2.75	0.02*	R post PHG	22 -44 -2	2.44	0.03*
				L post PHG	-22 -44 -2	2.41	0.05*
RTLE							
L Sup Temp G	-46 6 -14	2.75	0.003	R Rolandic O	52 -16 22	3.78	0.000
L Ant Cingulum	-2 42 -2	2.66	0.004	L Postcentral G	-32 -36 50	3.68	0.000
				R Mid Cingulum	18 -32 48	3.66	0.000
RTLE_Medial Temporal Lobe							
R ant HC	30 -2 -22	3.31	0.01*	L HC body	-22 -20 -16	2.74	0.03*
L ant HC	-34 -22 -16	2.52	0.05*	R post HC	22 -36 6	2.42	0.04*
Differences in Group Activations							
Controls> LTLE							
L medial OFC	-6 36 -16	2.84	0.003	R ant HC	30 -16 -22	2.60	0.03*
L amygdala	-30 2 -22	2.23	0.013	L ant PHG	-24 -12-32	2.84	0.016*
L ant PHG/HC	-22 -20 -20	2.13	0.015				
Controls>RTLE							
R med OFC	6 32 -10	3.57	0.000	R Inf Frontal G	32 6 -46	3.27	0.000
L ant Mid Temp G	-56 -12 -12	2.99	0.001	L PHG	-16 -18 24	2.09	0.018
				R ant HC	26 -12 -26	2.07	0.019
LTLE>Controls							
R Mid Frontal G	34 14 34	4.11	0.000	R Inf Frontal G	56 4 20	2.72	0.003
R Inf Frontal G	54 4 26	3.11	0.001				
R OFC	20 42 -18	2.45	0.005				
R ant HC	20 -12 -16	2.52	0.04*				
RTLE>Controls							
R Inf Frontal G	34 18 30	3.2	0.000	L Postcentral G	-48 -34 54	3.68	0.000
L Mid Frontal G	-30 32 16	2.75	0.003	R Postcentral G	58 -14 44	3.27	0.000

Supplementary Table 1

Region	Coordinate	Z score	P value	Region	Coordinate	Z score	P value
Longitudinal word encoding changes in Controls				Longitudinal face encoding changes in Controls			
**Time point two > Time point one**							
L Inf Frontal G	-60 8 18	4.27	0.000	L Inf Frontal G	-42 10 20	2.64	0.004
L Precentral G	-56 -4 32	3.87	0.000				
**Time point three > Time point one**							
L Inf Frontal G	-60 6 16	3.17	0.001	N/S			
**Time point three > Time point two**							
N/S				L OFC	-42 50 4	2.61	0.005
**Time point one > Time point two**							
N/S				R anterior HC	28 -12 -20	3.43	0.004*
				R OFC	42 22 -18	3.51	0.001
**Time point one > Time point three**							
N/S				R anterior HC	24 -18 -16	3.33	0.005*
**Time point two > Time point three**							
N/S				N/S			

Supplementary table 2

Correlation of change in memory score with fMRI activations						
**LTLE**						P value
Word Encoding	VL pre to postop1	Improvement	N/S			
		Decline	N/S			
	VL pre to postop2	Improvement	R anterior HC	26 -10 -20	2.78	0.03*
			R postcentral G	56 -8 34	3.2	0.001
			R anterior cingulum	6 16 28	2.97	0.001
		Decline	N/S			
	VL postop1 to postop2	Improvement	N/S			
		Decline	N/S			
Face Encoding	DL pre to postop1	Improvement	Anterior Cingulum	0 36 26	3.65	0.000
		Decline	N/S			
	DL pre to postop2	Improvement	N/S			
		Decline	N/S			
	DL postop1 to postop2	Improvement	N/S			
		Decline	N/S			
**RTLE**						
Face Encoding	DL pre to postop1	Improvement	N/S			
		Decline	L posterior HC	-30 -32 -10	3.94	0.006*
	DL postop1 to postop2	Improvement	L anterior HC/ amygdala	-30 2 -20	2.43	0.047*
			L insula	-32 2 0	3.08	0.001
			L Inf Frontal G	-60 4 18	3.00	0.001
			L Orbitofrontal C	-44 22 -16	2.99	0.001
		Decline	N/S			
	DL Pre to postop2	Improvement	L Orbitofrontal C	-40 56 -4	2.97	0.001
		Decline	N/S			
Word Encoding	V L pre to postop1	Improvement	N/S			
		Decline	L posterior HC	-32 -22 -10	2.47	0.049*
	VL pre to postop2	Improvement	L anterior HC/PHG	-28 -10 -28	2.55	0.046*
			L Orbitofrontal C	-18 56 -6	3.06	0.001
		Decline	R posterior HC	28 -32 -8	2.42	0.06*
	VL postop1 to postop2	Improvement	N/S			
	VL postop1 to postop2	Decline	R posterior HC	28 -32 -8	3.52	0.000*

Supplementary table 3
