# Supplementary material for: Memory network plasticity after temporal lobe resection: a longitudinal functional imaging study
Source: Brain. 2016 Jan 9;139(2):415–30. doi: 10.1093/brain/awv365 (PMC4805088; doi:10.1093/brain/awv365)
Supplement: Supplementary Data [file awv365_supplementary_data.zip › brain-2015-00632-File011.pdf]

Supplementary Table 1: Word and face encoding activations preoperatively in patients, and at the first scanning session in controls. *Medial temporal activations are shown corrected for multiple corrections, FWE (Family wise error) $p<0.05$ within a 10mm diameter sphere. L (left), R (right), Inf (inferior), Mid (middle), Sup (superior), Med (medial), G (gyrus), Ant (anterior), Post (posterior), HC (hippocampus), O (operculum).

Supplementary Table 2: Longitudinal changes in word and face encoding activations in controls. *Medial temporal activations are shown corrected for multiple corrections, FWE (Family wise error), $p<0.05$ within a 10mm diameter sphere. L (left), R (right), Inf (inferior), Mid G (gyrus), HC (hippocampus), OFC (Orbitofrontal cortex), N/S (no significant activations).

Supplementary Table 3: Correlation of change in verbal (VL) and design learning (DL) with word and face encoding activations at the corresponding time points in left (LTLE) and right temporal lobe epilepsy (RTLE) patients. *Medial temporal activations are shown corrected for multiple corrections, FWE (Family wise error), $p<0.05$ within a 10mm diameter sphere. Pre (preoperative), Postop1 (3 months post-operatively), postop2 (12 months post-operatively), L (left), R (right), Inf (inferior), G (gyrus), HC (hippocampus), PHG (parahippocampal gyrus), C (cortex), N/S (no significant activations).

Supplementary figure 1: Correlation of decline in design learning 3 months post-operatively in right temporal lobe epilepsy patients. The images show significant correlation of left posterior hippocampal activations 3 months post-operatively with decline in design learning 3 months post-operatively compared to preoperatively. Post (posterior), Preop (preoperative), Postop1 (3 months post-operatively).
