# Supplementary figures and images for: Memory network plasticity after temporal lobe resection: a longitudinal functional imaging study
Source: Brain. 2016 Jan 9;139(2):415–30. doi: 10.1093/brain/awv365 (PMC4805088; doi:10.1093/brain/awv365)

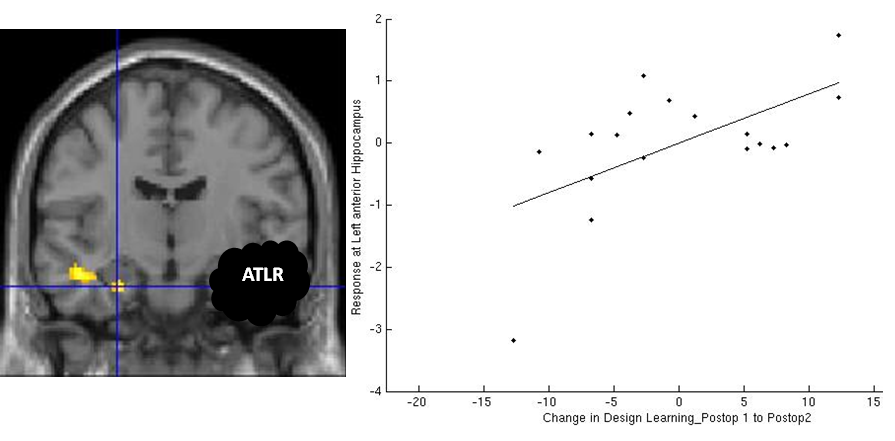

Supplement: Supplementary Data [file awv365_supplementary_data.zip › brain-2015-00632-File009.tif]
