# Supplementary material for: Memory network plasticity after temporal lobe resection: a longitudinal functional imaging study
Source: Brain. 2016 Jan 9;139(2):415–30. doi: 10.1093/brain/awv365 (PMC4805088; doi:10.1093/brain/awv365)
Supplement: Supplementary Table 1 [file brain_awv365_index.html]

Supplementary Data | Brain

## Supplementary Data

files

- Supplementary Data - pdf file
- Supplementary Data - pdf file
- Supplementary Data - tif file
